# The associations of continuity of care with inpatient, outpatient, and total medical care costs among older adults with urinary incontinence

**DOI:** 10.1186/s12913-023-09232-x

**Published:** 2023-04-06

**Authors:** Eunkyung Han, Wankyo Chung, Antonio Trujillo, Joel Gittelsohn, Leiyu Shi

**Affiliations:** 1 Johns Hopkins Bloomberg School of Public Health, Baltimore, MD USA; 2 Department of Public Health Science, Graduate School of Public Health, Seoul National University, Seoul, South Korea

**Keywords:** Continuity of care, Urinary incontinence, Health care costs, Primary health care

## Abstract

**Introduction:**

Urinary incontinence is a significant health problem with considerable social and economic consequences among older adults. The objective of this study was to investigate the financial impact of continuity of care (CoC) among older urinary incontinence patients in South Korea.

**Methods:**

We used the NHIS-Senior cohort patient data between January 1, 2010, and December 31, 2010. Patients who were diagnosed with urinary incontinence in 2010 were included. Operational definition of CoC included referrals, number of providers, and number of visits. A generalized linear model (GLM) with γ-distributed errors and the log link function was used to examine the relationship between health cost and explanatory variables. Additionally, we conducted a two-part model analysis for inpatient cost. Marginal effect was calculated.

**Results:**

Higher CoC was associated with a decrease in total medical cost (-0.63, *P* < .0001) and in outpatient costs (-0.28, *P* < .001). Higher Charlson Comorbidity Index (CCI) score was a significant predictor for increasing total medical cost (0.59, *P* < .0001) and outpatient cost (0.22, *P* < .0001). Higher CoC predict a reduced medical cost of $360.93 for inpatient cost (*P* = 0.044) and $23.91 for outpatient cost (*P* = 0.008) per patient.

**Conclusion:**

Higher CoC was associated with decrease in total medical costs among older UI patients. Policy initiatives to promote CoC of older UI patients in the community setting could lead to greater financial sustainability of public health insurance in South Korea.

**Supplementary Information:**

The online version contains supplementary material available at 10.1186/s12913-023-09232-x.

## Introduction

With the global aging trend, urinary incontinence (UI) is a significant health problem with considerable social and economic consequences among older adults [1]. The prevalence of UI reported in population-based studies globally ranges from 9.9% to 36.1% depending on the type of incontinence and age of population, [1] and is associated with decrease in activities in daily living (ADLs) and/or cognitive function [1–5]. In rapidly aging countries including South Korea, health cost burden among UI and overactive bladder (OAB) patients may also increase [6]. However, there are limited studies on effectively managing the burden of medical cost among older UI patients. Moreover, there is a lack of studies on the association between health costs and continuity of care (CoC) among this population.

CoC, defined as a patient’s attachment to their primary care practice, may reduce health care costs among older patients with chronic conditions. CoC characterizes the relationship between individual patients and their physicians over time and is expected to improve quality of care by fostering less expensive, less intensive medical care such as hospitalization [7–12]. Hollander et al. reported a clear inverse relationship between a patient’s attachment to a primary care practice and overall health care costs for hospital care, medical care, and drug prescription [8]. Hussey et al. reported a unit increase in CoC was associated with reduced health cost among patients with chronic diseases [9].

South Korea, forecasted to be the country with the largest percentage increase in the share of older adults in the world between 2019–2050 [10], is faced with the challenge of tackling increasing public health care needs as a result of population aging. In South Korea, Nam et al. (2019) reported that continuous care was associated with lower inpatient costs that led to reduction of total healthcare costs [11]. Oh et al. (2021) reported that higher CoC was associated with lower medical cost in patients diagnosed with chronic shoulder pain [12]. South Korean studies on CoC, mostly focused on diabetic and hypertensive patients, found that CoC was an effective factor to reduce mortality, hospitalization, and medical costs, while improving clinical outcomes such as comorbidity burden [11]. However, evidence for older UI patients in terms of the association between CoC and medical cost is scarce.

The objective of this study was to investigate financial impact of CoC among older UI patients in South Korea. In addition, we investigated whether previous long-term care (LTC) use had financial implication for the total medical spending.

## Methods

### Data source

In this study, we used administrative health claims data from the South Korean National Health Insurance Service (NHIS) Senior Cohort. NHIS-Senior cohort is a Korean nationwide retrospective administrative data cohort, composed of older adults aged 60 years and over in 2002 [13]. It consists of 558,147 people selected by 10% simple random sampling method from a total of 5.5 million subjects aged 60 years and over in the National Health Information Database of South Korea [13]. The cohort was followed up through 2015 for all subjects, except for those who were deceased [13]. All patient information and records were provided in de-identified form of person specific number and organization code.

### Study design and population

We used NHIS-Senior cohort patient data between January 1, 2010, and December 31, 2010. This was the first full-year dataset with several refined data for the study population after the public LTC insurance system for older adults was implemented in South Korea. The 2010 dataset is still relevant today since there is little change in health insurance policy and primary care delivery system in Korea compared to 2010. The dataset included patients who were diagnosed as UI (KCD N328, N329, N393, N394, R32) at least once between January 1, 2010 to December 31, 2010 (*n* = 32,871). Patients with prior diagnosis of disease during the preceding two years (between 2008–2009) were excluded (*n* = 27,295). In total, 5,576 patients were included in the final analysis. Therefore, we considered the data of 2010 was the first and most expansive data which was accessible for our study aims.

### Measures of study variables

#### Episode of Care (EoS)

An EoS is defined as a set of services provided to treat a clinical condition or procedure [14, 15]. In this study, we operationally defined EoS as all NHI services provided during a patient’s hospitalization period. As a result, we constructed an episode of care to avoid overestimation due to the number of hospitalizations. Since our analysis was based on administrative claim data, there were separate hospitalization claims even if the patient was treated with the same disease or treatment. To address this, if the difference between the discharge date and the subsequent hospitalization date of the hospitalization claim was less than 1 day, we considered this as the same treatment and grouped into a single episode [16].

#### Continuity of Care (CoC)

In this study, ambulatory care continuity was measured using CoC index [17]. The CoC index reflected the distribution of visits to different providers from different healthcare institutions. CoC index was influenced by both the total number of providers and the total number of visits [17, 18]. Subjects with three or less visits per year were excluded. In this study, the value of CoC which ranges from 0 to 1 was converted into categorical data to facilitate comparison between the higher CoC group and the lower CoC group. A patient was described as having a low continuity if CoC was < 0.75 and high continuity if CoC was ≥ 0.75. This categorization was consistent with previous studies using CoC index [18–21]. The formula for the CoC index [17] is as follows:

$$\mathrm{coc}= \frac{\sum {n}_{j}^{2}-N}{N\left(N-1\right)}$$

where *N* = total number of visits, *n* = number of visits to provider, and j = specific provider.

In sensitivity analysis, we constructed a continuous CoC Index (CoCI) without cut-off of 0.75, to test whether our findings were consistent. We tested for outpatient, inpatient, and total medical costs using CoCI with the same study population.

#### Outcome variables

As a single-payer system, South Korean government covers health cost via a single national health insurer, the National Health Insurance Corporation (NHIC). This database only captures the NHI cost. Therefore, total health expenditures included both the payer’s (NHIC) and patients’ (out-of-pocket) medical costs.

Total medical costs included a combination of inpatient and outpatient costs incurred between January 1, 2010, and December 31, 2010. Each patient’s total inpatient and outpatient costs were derived and used as inpatient cost and outpatient cost, respectively. For each patient, the cost value included both the payer’s (South Korean national health insurance) and patient’s out-of-pocket costs.

In addition, the total amount of medical costs for each patient in the previous year was calculated and used. The previous year’s costs (health expenses incurred during Jan 1, 2009, to Dec 31, 2009) were derived from the data. The previous year’s medical costs measure was recoded as 0 to 25% (low spender), 25% to 50% (low to median spender), 50% to 75% (median to high spender), and 75% to 100% (high spender) with higher percentage indicating higher spending among our study population.

#### Other covariates

Other explanatory covariates included sociodemographic factors (age, gender, disability, income, insurance status, region), Charlson’s Comorbidity Index (CCI) score, and body mass index (BMI).

Total income was originally reported as medical aid or 1^st^ to 10^th^ decile in total income level. The income level was categorized into four groups. Health insurance status indicated whether the patient was enrolled in the South Korean national health insurance or medical aid. The region of a patient’s residence was coded as metropolitan areas (Seoul, Gyunggi, Incheon) or non-metropolitan areas (elsewhere).

The patient’s CCI score referred to the weighted number of comorbid conditions the patient had been diagnosed based on the methods by Quan et al. [22]. In this study, CCI score (0, 1, 2, or 3) was calculated to measure patient’s burden of disease [23, 24]. CCI scores 3 and higher were coded as 3 for the sake of analysis.

Each patient’s weight in kilograms was divided by the square of height in meters and was used as a continuous variable. Weight and height data were collected from health screening results prior to 2010. If a person had multiple records of height and weight, data collected when UI was first diagnosed was used.

If a patient used LTC service (whether home-based or facility-based) in the previous year, the variable LTC use was coded as 1. Otherwise, LTC use was coded as 0.

### Statistical analysis

A generalized linear model (GLM) with γ-distributed errors and the log link function was used to examine the relationship between health cost and explanatory variables. Our outcome variables, total medical costs, outpatient costs, and inpatient costs in 2010, were right skewed, therefore we used GLM instead of the traditional ordinary least square (OLS) model. In addition, inpatient cost had many zero values, so we conducted a two-part model analysis and marginal effect calculation for inpatient cost. Confounding variables included CCI score, cost from previous year, age, income, disability, gender, residence (in metropolitan area), BMI, and LTC service use. Statistical significance was set at *p* < 0.05. All analysis were conducted using SAS Enterprise Guide 7.1 and STATA 15.

## Results

A total of 5,576 patients were included in the final analysis. Mean age was 74.34 and the proportion of male was 22.97% (*n* = 1,281). Patients in the study population were mostly women, who were equal to or older than 75 years old (64.2%). Of the total number of patients, 78.6% had at least one chronic condition. They were mostly (86.0%) enrolled in a national health insurance (NHI) service. For income status, 19.8% of the patients were at the lowest quartile and 26.8% the highest quartile. In 2010, among all patients, mean total medical costs were $963.70, mean outpatient costs $87.61 and mean inpatient costs $876.09. Only 3.6% of all patients had LTC use in the previous year (2009) (See Table 1).

**Table 1:** Characteristics of the study sample

N(%) or Mean (SD)	Total	Female	Male	*P* value
Total medical cost^a^	963.70 (2329.18)	981.63 (2369.52)	903.59 (2188.29)	0.27
Min/Max	5.16/38741.57	5.16/38741.57	8.42/29029.34	
Outpatient cost^a^	87.61 (194.70)	83.79 (200.05)	100.41 (175.06)	0.004
Min/Max	5.16/5761.65	5.16/5761.65	8.42/2587.49	
Inpatient cost^a^	876.09 (2310.47)	897.84 (2349.64)	803.13 (2173.31)	0.17
Min/Max	0.00/38716.69	0.00/38716.69	0.00/28990.93	
BMI	24.52 (3.30)	24.75 (3.39)	23.84 (2.92)	< .0001
Min/Max	13.80/43.90	13.80/43.90	15.50/33.50	
CoC				0.81
Low	5264 (94.40)	4053 (94.37)	1211 (94.54)	
High	312 (5.60)	242 (5.63)	70 (5.46)	
CCI score				0.07
0	1192 (21.38)	918 (21.37)	274 (21.39)	
1	1663 (29.82)	1315 (30.62)	348 (27.17)	
2	1224 (21.95)	936 (21.79)	288 (22.48)	
3 +	1497 (26.85)	1126 (26.22)	371 (28.96)	
Previous medical cost				0.90
Lowest	1103 (19.84)	818 (19.09)	285 (22.37)	
Lower	1440 (25.90)	1112 (25.94)	328 (25.75)	
Higher	1528 (27.48)	1213 (28.30)	315 (24.73)	
Highest	1489 (26.78)	1143 (26.67)	346 (27.16)	
Previous LTC use				< .0001
No	5374 (96.38)	4111 (95.72)	1263 (98.59)	
Yes	202 (3.62)	184 (4.28)	18 (1.41)	
Age				< .0001
< 75 years	1999 (35.85)	1612 (37.53)	387 (30.21)	
≥ 75 years	3577 (64.15)	2683 (62.47)	894 (69.79)	
Insurance type				< .0001
National Health Insurance	4794 (85.98)	3615 (84.17)	1179 (92.04)	
Medical aid	782 (14.02)	680 (15.83)	102 (7.96)	
Income				< .0001
Low	1749 (31.37)	1438 (33.48)	311 (24.28)	
Lower-Middle	925 (16.59)	694 (16.16)	231 (18.03)	
Upper-Middle	878 (15.75)	669 (15.58)	209 (16.32)	
High	2024 (36.30)	1494 (34.78)	530 (41.37)	
Region				0.10
Other area	3394 (60.87)	2639 (61.44)	755 (58.94)	
Metropolitan area	2182 (39.13)	1656 (38.56)	526 (41.06)	
Disability				0.74
No	5528 (99.14)	4259 (99.16)	1269 (99.06)	
Yes	48 (0.86)	36 (0.84)	12 (0.94)	

*Abbreviations*: *BMI* Body Mass Index, *CoC* Continuity of Care, *CCI* Charlson Comorbidity Index, *LTC* Long-Term Care

^a^All currency was converted to US dollars ($) in 2010. See reference [25] for conversion criteria and resource

Regression coefficients between medical cost and each predictor variable are shown in Table 2. High CoC was associated with a decrease in total medical costs (-0.63, *P* < 0.0001) and outpatient costs (-0.28, *P* = 0.0002). Having higher CCI score was a significant predictor for increasing total medical costs (0.59, *P* < 0.0001) and outpatient costs (0.23, *P* < 0.0001). Compared to those at the lowest 25% quartile in total medical costs in the previous year, those in the higher quartile groups also incurred significantly higher total medical costs (25%-50% coefficient 0.20, 50%-75% coefficient 0.52, > 75% coefficient 0.68, all *P* < 0.05) and outpatient medical costs (25%-50% coefficient 0.09, 50%-75% coefficient 0.18, > 75% coefficient 0.29, all *P* < 0.05) for the study year. In other word, more spending in the previous year was significantly associated with more spending in the study year. Compared to the lowest income group, higher income groups incurred higher outpatient costs (in the 9^th^-10^th^ group, coefficient 0.23, *P* < 0.0001) but the impact was not significant in total medical costs. Those residing in metropolitan area had significantly lower total medical costs (-0.11, *P* < 0.05). Age over 75 years (-0.17, *P* < 0.0001) and being female (-0.20, *P* < 0.0001) were associated with decrease in outpatient medical costs but not in total medical costs. A 1-unit increase in BMI was significantly associated with increase in total medical costs (0.02, *P* < 0.05), but the effect was not significant in outpatient medical costs. LTC use in the previous year was not significantly associated with outpatient or total medical costs.

**Table 2:** Predictors of medical cost among older UI patients

Factors	Total medical cost				Outpatient medical cost			
	Coef	95% CI		*P* value	Coef	95% CI		*P* value
		LB	UB			LB	UB	
CoC (reference: low CoC)								
High	-0.63	-0.89	-0.37	< .0001	-0.28	-0.43	-0.13	0.0002
CCI score (reference: 0)								
1	0.27	0.13	0.42	0.0002	-0.01	-0.09	0.07	0.86
2	0.34	0.19	0.50	< .0001	0.07	-0.01	0.17	0.09
3 +	0.59	0.44	0.75	< .0001	0.22	0.13	0.32	< .0001
Previous medical cost (reference: lowest)								
Lower ($299.83 to $670.88)	0.20	0.04	0.36	0.01	0.09	0.00	0.18	0.04
Higher ($670.89 to $1489.37)	0.52	0.37	0.68	< .0001	0.18	0.09	0.27	< .0001
Highest (≥ $1489.38)	0.67	0.52	0.84	< .0001	0.29	0.20	0.39	< .0001
Previous LTC use (reference: no use)								
Yes	-0.10	-0.44	0.24	0.55	0.01	-0.19	0.20	0.95
Age (reference: < 75 years)								
age ≥ 75	0.02	-0.09	0.14	0.64	-0.16	-0.23	-0.10	< .0001
Gender (reference: male)								
Female	0.01	-0.11	0.13	0.84	-0.20	-0.27	-0.13	< .0001
Income, n(%) (reference: low)								
Lower-Middle	0.01	-0.15	0.16	0.91	0.09	0.01	0.19	0.04
Upper-Middle	0.12	-0.04	0.28	0.15	0.05	-0.04	0.15	0.23
High	-0.09	-0.23	0.04	0.16	0.22	0.15	0.30	< .0001
Region (reference: other area)								
Metropolitan area	-0.10	-0.21	-0.01	0.048	0.01	-0.06	0.06	0.98
Disability (reference: no disability)								
Yes	0.61	-0.06	1.28	0.07	-0.35	-0.73	0.03	0.07
BMI	0.02	0.00	0.04	0.02	0.01	-0.01	0.02	0.08

*Abbreviations*: *Coef* Coefficient estimate in the regression, *CI* Confidence Interval, *LB* Lower Bound, *UB* Upper Bound, *CoC* Continuity of Care, *CCI* Charlson Comorbidity Index, *LTC* Long-Term Care, *BMI* Body Mass Index

We performed a separate two-part model analysis for inpatient costs. The first part was about the use of inpatient services (See Table 3). Higher CoC was associated with decreased hospitalization (-0.44, *P* < 0.0001), which had a bigger effect than other factors as shown in the magnitude of the coefficients. Higher income was associated with lower hospitalization. Having multiple weighted comorbidities (0.14 for CCI score 1; 0.20 for CCI score 2; 0.37 for having equal or more than CCI score 3, all *P* < 0.05) was associated with increased hospitalization. The second part analysis (See Table 4) using GLM showed that being in the upper-middle income group, compared to the lowest income group, was associated with increase in inpatient costs (0.25, *P* = 0.04).

**Table 3:** Inpatient medical cost predictors among older UI patients using two-part model (first part analysis using probit model)

Factors	Coef	95% CI		*P* value
		LB	UB	
First part (probit)				
CoC (reference: low CoC)				
High	-0.44	-0.68	-0.22	< .0001
CCI score ( reference: 0)				
1	0.14	0.02	0.26	0.02
2	0.20	0.08	0.34	0.002
3 +	0.37	0.25	0.50	< .0001
Previous medical cost (reference: lowest)				
Lower ($299.83 to $670.88)	0.14	0.01	0.27	0.03
Higher ($670.89 to $1489.37)	0.31	0.19	0.44	< .0001
Highest (≥ $1489.38)	0.53	0.40	0.66	< .0001
Previous LTC use (reference: no use)				
Yes	0.10	-0.14	0.36	0.39
Age (reference: < 75 years)				
age ≥ 75	0.06	-0.02	0.15	0.14
Gender (reference: male)				
Female	-0.07	-0.17	0.01	0.11
Income, n(%) (reference: low)				
Lower-Middle	-0.12	-0.26	-0.01	0.04
Upper-Middle	-0.15	-0.28	-0.02	0.02
High	-0.12	-0.23	-0.01	0.02
Region (reference: other area)				
Metropolitan area	-0.07	-0.16	0.01	0.09
Disability (reference: no disability)				
Yes	0.25	-0.23	0.73	0.30
BMI	0.01	-0.01	0.01	0.33

*Abbreviations*: *Coef* Coefficient estimate in the regression, *CI* Confidence Interval, *LB* Lower Bound, *UB* Upper Bound, *CoC* Continuity of Care, *CCI* Charlson Comorbidity Index, *LTC* Long-Term Care, *BMI* Body Mass Index

**Table 4:** Inpatient medical cost predictors among older UI patients using two-part model (second part analysis using GLM)

Factors	Coef	95% CI		*P* value
		LB	UB	
Second part (GLM)				
CoC (reference: low CoC)				
High	0.05	-0.30	0.40	0.78
CCI score (reference: 0)				
1	0.14	-0.10	0.36	0.24
2	0.12	-0.10	0.34	0.29
3 +	0.19	-0.03	0.42	0.09
Previous medical cost (reference: lowest)				
Lower ($299.83 to $670.88)	-0.04	-0.17	0.36	0.76
Higher ($670.89 to $1489.37)	0.09	-0.23	0.29	0.47
Highest (≥ $1489.38)	0.03	-0.03	0.35	0.84
Previous LTC use (reference: no use)				
Yes	-0.10	-0.39	0.20	0.52
Age (reference: < 75 years)				
age ≥ 75	0.02	-0.13	0.14	0.90
Gender (reference: male)				
Female	0.15	-0.01	0.30	0.05
Income, n(%) (reference: low)				
Lower-Middle	0.16	-0.03	0.35	0.11
Upper-Middle	0.25	0.01	0.49	0.04
High	-0.03	-0.19	0.13	0.69
Region (reference: other area)				
Metropolitan area	-0.02	-0.15	0.14	0.95
Disability (reference: no disability)				
Yes	0.35	-0.35	1.06	0.32
BMI	0.02	-0.003	0.04	0.12

*Abbreviations*: *GLM* Generalized Linear Model, *Coef* Coefficient estimate in the regression, *CI* Confidence Interval, *LB* Lower Bound, *UB* Upper Bound, *CoC* Continuity of Care, *CCI* Charlson Comorbidity Index, *LTC* Long-Term Care, *BMI* Body Mass Index

Using the estimates from the generalized linear model analyses, we estimated the expected overall medical cost avoidance that increase of CoC would have impacted. Higher CoC could predict a $360.93 reduction in inpatient costs (*P* = 0.044); $23.91 reduction in outpatient costs (*P* = 0.008); and $569.80 reduction in total medical costs (*P* = 0.002) (See Table 5).

**Table 5:** Estimated marginal effect of CoC among older UI patients (US dollars)^a^

Cost category	Marginal Effect	95% CI		*P* value
		LB	UB	
Inpatient cost	-360.93	-713.12	-8.89	0.044
Outpatient cost	-23.91	-41.43	-6.38	0.008
Total medical cost	-569.80	-930.28	-209.33	0.002

*Abbreviations*: *CI* Confidence Interval, *LB* Lower Bound, *UB* Upper Bound, *CoC* Continuity of Care

^a^All currency was converted to US dollars ($) in 2010. See reference [25] for conversion criteria and resource

In sensitivity analyses, a CoC variable without cut-off (CoCI) yielded similar results (See Supplementary materials). High CoCI was associated with decrease in total medical cost (-0.82, *P* < 0.0001), outpatient medical cost (-0.60, *P* < 0.0001) and chance of hospitalization in probit model (-0.87, *P* = 0.00). The magnitude of coefficients was greater than that in the findings using CoC variable with cutoff point at 0.75.

## Discussion

To our knowledge, this is the first study to investigate the association between CoC and medical costs among older UI patients, using the South Korean National Health Insurance Service cohort database. We found that maintaining a higher level of CoC, compared to maintaining a lower level, led to a decrease in outpatient, inpatient, and total medical costs. This result is consistent with other studies that showed similar findings in terms of the inverse association between CoC and medical costs [10–12, 26–31]. This finding also holds true for various countries with distinct healthcare systems [32].

This study showed that the major driver of cost-saving was due to reduced hospitalization, as Chen et al. [31] also reported. Similar findings are evident from other studies [27, 29] which used South Korean health insurance claims data. Examining various sources of cost drivers in older adults is important, as Lei et al. [28] reported that a significant segment of cost reduction was in institutional care.

Controlling for other factors, we found that enhancing outpatient CoC in the study participants could have resulted in an estimated reduction of $569.80 in total medical costs for each patient annually. Savings achieved on inpatient costs were greater than on outpatient costs. Our study showed that CoC was a potentially modifiable factor in the health care for older patients with multimorbidity [33]. The policy implication of these results suggested that the South Korean government could consider enhancingCoC not only to overcome ambulatory care fragmentation and unnecessary medical visits, but also as a strategy to achieve cost-saving [34, 35].

Our study’s results did not show a statistically significant decrease in inpatient costs among patients with a higher level of CoC. This implies that above certain threshold of CoC, more primary care did not achieve more cost reduction (i.e., negative marginal effects on annual inpatient costs). However, we did see a statistically significant association between higher CoC and lower odds of incurring hospitalization. Several other studies about patients with dementia or cancer reported that higher level of CoC was associated with decreases in inpatient, outpatient, and total medical costs [27–31, 36, 37]. The difference in results between our study and other studies may be due to differences in health problems studied (i.e., UI versus dementia or cancer).

Our study findings may also be used as the basis for calculating the magnitude of the financial incentives required to foster patient participation in outpatient settings. The financial benefits of greater CoC can be offered to patients as incentives to change their healthcare use behavior. Based on the CoC index formula used in this and other studies [11, 12, 17, 18, 27, 28, 31], a benchmark for high CoC could be set, which may assist some types of public programs aimed at reducing avoidable hospitalization among community-dwelling patients with chronic conditions, such as the Community-Based Chronic Disease Management Program launched by the South Korean government [38].

LTC use in the previous year was associated with a decrease in inpatient costs but an increase in outpatient costs, although neither effect was statistically significant. Since other studies reported a positive association between LTC use and acute medical use, as well as between LTC costs and medical costs [39–41], the relationship between LTC utilization and medical spending needs further investigation. In addition, further studies are required of specific older patient groups with chronic conditions other than UI (e.g., dementia, frailty, or polypharmacy) [42, 43].

A one-unit increase in BMI increased total medical costs; however, we did not find that normal weight (BMI between 18 and 25) was a statistically significant factor in decreasing total, outpatient, or inpatient costs. This finding may suggest that the severity of various kinds of chronic condition affects medical costs more than BMI per se does.

### Limitations

Our study has several limitations. First, this study focused on individuals with UI; therefore, it is difficult to generalize our results to all older adults with multiple chronic conditions. However, UI is a common geriatric condition with many underreported cases. Thus, we think that this study still presents valuable knowledge regarding the impact of LTC on older adults.

Second, the primary database of this study (the NHIS Senior Cohort database) was mainly built on data established for billing purpose; therefore, clinical details could be deficient. Administrative claims data could be limited in its accuracy with regard to diagnoses. Furthermore, we could not capture out-of-pocket medical expenditures that were not collected by the original dataset.

Third, due to limitation of the database, the measure of CoC was less than ideal. It is necessary to enhance the qualitative characteristics of the continuous provision of care [44], which include aspects such as exhibiting a higher level of respectfulness and trustworthiness [45], enhancing resource management and monitoring systems, [46] and ensuring the quality of care given by medical providers, which are beyond the current method of measuring CoC on the basis of quantitative factors such as the number of medical visits. Future research could also collect data that examine types of chronic conditions likely associated with older adults with UI and to which extent they affect ADL and cognitive function. Furthermore, ways to improve CoC for institutionalized older adults, as well as for older adults who reside in the community, should be further examined.

## Conclusion

CoC was associated with decrease in total medical costs among older UI patients. Controlling for other factors, we found that increasing CoC in this patient population could have resulted in an estimated cost avoidance of $569.80 for total medical costs for each patient annually. Policy initiatives to promote CoC of older UI patients in the community setting could lead to greater financial sustainability for public health insurance in South Korea.

## Supplementary Information

**Additional file 1.**

## Data Availability

The dataset analyzed for this study is only available when permitted upon application due to the National Health Insurance Corporation’s data regulations related to the concerns of confidentiality for study subjects. Access to the dataset should be requested to the National Health Insurance Sharing Service of South Korea (nhiss.nhis.or.kr).

## Abbreviations

CCI: Charlson Comorbidity Index
CoC: Continuity of Care
GLM: Generalized Linear Model
LTC: Long-Term Care
NHIC: National Health Insurance Corporation
OLS: Ordinary Least Square
UI: Urinary Incontinence
