# Supplementary material for: The associations of continuity of care with inpatient, outpatient, and total medical care costs among older adults with urinary incontinence
Source: BMC Health Serv Res. 2023 Apr 6;23:344. doi: 10.1186/s12913-023-09232-x (PMC10080744; doi:10.1186/s12913-023-09232-x)
Supplement: Supplementary file 1 — Additional file 1. [file 12913_2023_9232_MOESM1_ESM.docx]

**Supplementary Material**

Table S1. Predictors of total medical cost among older urinary incontinence patients

| Factors | Total medical cost | | | | Outpatient medical cost | | | |
| --- | --- | --- | --- | --- | --- | --- | --- | --- |
|  | Coef. | 95% CI (L) | 95% CI (R) | P value | Coef. | 95% CI (L) | 95% CI (R) | P value |
| CoCI | -0.82 | -1.15 | -0.50 | <.0001 | -0.60 | -0.78 | -0.41 | <.0001 |
| CCI score (reference: 0) | | | | | | | | |
| 1 | 0.26 | 0.09 | 0.42 | 0.0023 | -0.03 | -0.13 | 0.05 | 0.4349 |
| 2 | 0.25 | 0.07 | 0.43 | 0.0059 | 0.02 | -0.08 | 0.13 | 0.6446 |
| 3+ | 0.55 | 0.38 | 0.73 | <.0001 | 0.21 | 0.11 | 0.32 | <.0001 |
| Previous medical cost (reference: lowest) | | | | | | | | |
| Lower | 0.42 | 0.24 | 0.60 | <.0001 | 0.15 | 0.04 | 0.25 | 0.0055 |
| Higher | 0.73 | 0.55 | 0.90 | <.0001 | 0.28 | 0.17 | 0.38 | <.0001 |
| Highest | 0.87 | 0.69 | 1.05 | <.0001 | 0.35 | 0.24 | 0.45 | <.0001 |
| Previous LTC use (reference: no use) | | | | | | | | |
| Yes | -0.07 | -0.45 | 0.30 | 0.6900 | 0.00 | -0.21 | 0.21 | 0.9919 |
| Age (reference: <75 years) | | | | | | | | |
| Age>75 | -0.00 | -0.13 | 0.12 | 0.9474 | -0.21 | -0.30 | -0.14 | <.0001 |
| Gender (reference: male) | | | | | | | | |
| Female | -0.10 | -0.27 | 0.05 | 0.1914 | -0.25 | -0.34 | -0.16 | <.0001 |
| Income, n(%) reference: low) | | | | | | | | |
| Lower-Middle | 0.03 | -0.14 | 0.21 | 0.7039 | 0.12 | 0.02 | 0.23 | 0.0168 |
| Upper-Middle | -0.16 | -0.35 | 0.02 | 0.0849 | 0.09 | -0.01 | 0.20 | 0.0761 |
| High | -0.09 | -0.24 | 0.06 | 0.2364 | 0.23 | 0.14 | 0.32 | <.0001 |
| Region (reference: other area) | | | | | | | | |
| Metropolitan area | -0.11 | -0.24 | 0.00 | 0.0701 | -0.06 | -0.14 | 0.00 | 0.0585 |
| Disability (reference: no disability) | | | | | | | | |
| Yes | 0.80 | 0.02 | 1.58 | 0.0438 | -0.40 | -0.84 | 0.04 | 0.0763 |
| BMI | 0.02 | 0.00 | 0.04 | 0.0149 | 0.00 | -0.00 | 0.01 | 0.2379 |

Abbreviations: Coef, Coefficient estimate in the regression; CI, Confidence Interval; LB, Lower Bound; UB, Upper Bound; CoCI, Continuity of Care Index; CCI, Charlson Comorbidity Index; LTC, Long-Term Care; BMI, Body Mass Index.

Table S2. Inpatient medical cost predictors among older urinary incontinence patients using two-part model

| Factors | First part (probit) | | | | Second part (GLM) | | | |
| --- | --- | --- | --- | --- | --- | --- | --- | --- |
|  | Coef. | 95% CI (L) | 95% CI (R) | P value | Coef. | 95% CI (L) | 95% CI (R) | P value |
| CoCI | -087 | -1.14 | -060 | 0.00 | 0.44 | 0.00 | 0.88 | 0.05 |
| CCI score (reference: 0) | | | | | | | | |
| 1 | 0.15 | 0.15 | 0.29 | 0.03 | 0.10 | -0.15 | 0.34 | 0.45 |
| 2 | 0.16 | 0.01 | 0.31 | 0.04 | 0.07 | -0.18 | 0.31 | 0.59 |
| 3+ | 0.37 | 0.22 | 0.51 | 0.00 | 0.13 | -0.12 | 0.39 | 0.30 |
| Previous medical cost (reference: lowest) | | | | | | | | |
| Lower | 0.15 | 0.00 | 0.31 | 0.06 | 0.14 | -0.10 | 0.39 | 0.25 |
| Higher | 0.31 | 0.16 | 0.47 | 0.00 | 0.33 | 0.10 | 0.55 | 0.00 |
| Highest | 0.52 | 0.36 | 0.67 | 0.00 | 0.22 | -0.01 | 0.45 | 0.10 |
| Previous LTC use (reference: no use) | | | | | | | | |
| Yes | 0.20 | -0.07 | 0.48 | 0.15 | -0.11 | -0.41 | 0.18 | -0.46 |
| Age (reference: <75 years) | | | | | | | | |
| Age>75 | 0.05 | -0.05 | 0.15 | 0.35 | 0.00 | -0.15 | 0.15 | 0.99 |
| Gender (reference: male) | | | | | | | | |
| Female | -0.15 | -0.27 | -0.03 | 0.01 | 0.11 | -0.05 | 0.28 | 0.19 |
| Income, n(%) reference: low) | | | | | | | | |
| Lower-Middle | -0.13 | -0.27 | 0.02 | 0.10 | 0.15 | 0.07 | 0.37 | 0.17 |
| Upper-Middle | -0.20 | -0.35 | -0.05 | 0.01 | -0.05 | -0.28 | 0.17 | 0.64 |
| High | -0.11 | -0.23 | 0.00 | 0.07 | -0.04 | -0.22 | 0.14 | 0.63 |
| Region (reference: other area) | | | | | | | | |
| Metropolitan area | -0.06 | -0.16 | 0.04 | 0.27 | -0.05 | -0.21 | 0.11 | 0.54 |
| Disability (reference: no disability) | | | | | | | | |
| Yes | 0.57 | -0.01 | 1.14 | 0.05 | 0.33 | -0.34 | 1.00 | 0.33 |
| BMI | 0.01 | -0.01 | 0.02 | 0.42 | 0.02 | -0.00 | 0.04 | 0.06 |

Abbreviations: GLM, Generalized Linear Model; Coef, Coefficient estimate in the regression; CI, Confidence Interval; LB, Lower Bound; UB, Upper Bound; CoCI, Continuity of Care Index; CCI, Charlson Comorbidity Index; LTC, Long-Term Care; BMI, Body Mass Index.
